# Placental defects lead to embryonic lethality in mice lacking the Formin and PCP proteins Daam1 and Daam2

**DOI:** 10.1371/journal.pone.0232025

**Published:** 2020-04-30

**Authors:** Masa-aki Nakaya, Kristibjorn Orri Gudmundsson, Yuko Komiya, Jonathan R. Keller, Raymond Habas, Terry P. Yamaguchi, Rieko Ajima

**Affiliations:** 1 Cancer and Developmental Biology Laboratory, Center for Cancer Research, National Cancer Institute-Frederick, National Institutes of Health, Frederick, Maryland, United State of America; 2 Mouse Cancer Genetics Program, Center for Cancer Research, National Cancer Institute-Frederick, National Institutes of Health, Frederick, Maryland, United State of America; 3 Department of Biology, College of Science and Technology, Temple University, Philadelphia, Pennsylvania, United State of America; Laboratoire de Biologie du Développement de Villefranche-sur-Mer, FRANCE

## Abstract

The actin cytoskeleton plays a central role in establishing cell polarity and shape during embryonic morphogenesis. Daam1, a member of the Formin family of actin cytoskeleton regulators, is a Dvl2-binding protein that functions in the Wnt/Planar Cell Polarity (PCP) pathway. To examine the role of the Daam proteins in mammalian development, we generated *Daam*-deficient mice by gene targeting and found that *Daam1*, but not *Daam2*, is necessary for fetal survival. Embryonic development of *Daam1* mutants was delayed most likely due to functional defects in the labyrinthine layer of the placenta. Examination of *Daam2* and *Daam1/2* double mutants revealed that *Daam1* and *Daam2* are functionally redundant during placental development. Of note, neural tube closure defects (NTD), which are observed in several mammalian PCP mutants, are not observed in *Wnt5a* or *Daam1* single mutants, but arise in *Daam1;Wnt5a* double mutants. These findings demonstrate a unique function for *Daam* genes in placental development and are consistent with a role for *Daam1* in the Wnt/PCP pathway in mammals.

## Introduction

The actin cytoskeleton plays a central role in the morphogenesis of the mammalian embryo by controlling cell shape, division, polarity and movement. Previous studies revealed that Rho-GTPases are important regulators of the actin cytoskeleton [1, 2] and are essential for embryogenesis because loss of the Rho family members Rac1 or Cdc42 leads to early embryonic lethality [3, 4]. One signaling pathway known to control the activity of Rho family proteins during mammalian development is the Wnt/Planar Cell Polarity (PCP) pathway [5]. Dishevelled2 (Dvl2) is a cytoplasmic phosphoprotein that has common roles in transducing Wnt signals. Dvl2 possesses three conserved functional domains, the N-terminal DIX domain, a central PDZ domain, and a C-terminal DEP domain (reviewed in [6, 7]). The DIX domain is essential for transducing canonical Wnt signals through the Wnt/βcatenin pathway, whereas the PDZ and DEP domains function in the Wnt/PCP pathway [8–12].

Core components of the PCP pathway, which include the aforementioned Dvl, as well as the *frizzled* Wnt receptors, the transmembrane protein *Van Gogh*, the LIM-domain containing protein *prickle*, the G-protein coupled protein *flamingo*, and the ankyrin repeat-containing protein *diego*, were first identified in *Drosophila* where they were shown to control the direction of cells in the wing epidermis and eye. Establishment of PCP depends on the asymmetric localization of the PCP core components in these cells (Reviewed in [13]). Rho and Rac function downstream of these PCP core genes to control tissue polarity. In *Xenopus* and zebrafish, RhoA, Rac1 and Cdc42, along with the PCP pathway, control the convergence and extension (CE) movements responsible for the cell intercalations in the midline that drive the extension of the embryonic anterior-posterior (A-P) axis (reviewed in [5, 14–17]). Asymmetric localization of PCP core proteins was also observed in the cells undergoing the CE movements [18, 19].

Mice with mutations in core PCP genes exhibit a common set of phenotypes, including defects in the cochlea, polarity of multiciliated cells, Left-Right (LR) axis formation, A-P axis elongation, neural tube closure, and cardiac outflow tract septation [20–36]. Asymmetric localization of core PCP proteins in hair cells of the cochlea, multiciliated cells, and node cells has been reported. Disruption of these PCP proteins perturbed the orientation of the cells in the inner cochlea and multiciliated cells, or cilia position in the node in a fashion that is analogous to the PCP defects observed in the fly wing [22, 23, 27–29, 31–33, 37–39]. On the other hand, the mammalian A-P axis and neural tube closure defects are more similar to the CE movement defects observed when the PCP pathway is perturbed in *Xenopus* and zebrafish. These data suggest that the Wnt/PCP pathway has adapted to function in several different developmental contexts in diverse organisms.

These functional adaptations may have arisen from the regulation of different Rho GTPase effectors in a given cell type or tissue (reviewed in [40, 41]). Daam1 is a member of the Formin subfamily of Rho GTPase effectors that was originally identified as a Dvl2 binding protein [42]. Formins contain a GTPase binding domain (GBD) at the N-terminus, and the Formin homology domains (FH) 1 and 2 at the C-terminus of the protein. Formins are auto-inhibited by interactions between the N-terminus diaphanous inhibitory domain (DID) and the C-terminus diaphanous autoregulatory domain (DAD). Binding of Rho GTPases to the GBD domain alleviates auto-inhibition, exposing the FH2 functional domain to increase nucleation of the actin cytoskeleton (reviewed in [43–45]). The N-terminal region of the Daam1 protein containing the GBD and DID domains has dominant negative activity, whereas the C-terminal region of Daam1, which contains the FH2 domain, is constitutively active [42, 46]. In mammals, a second member of this highly conserved family, *Daam2*, exhibits both distinct and overlapping expression patterns with Daam1 during early mouse development [47]. Daam1 interacts with the PDZ and DEP domains of Dvl2 to control CE in *Xenopus* and zebrafish, consistent with a role of Daam1 in the Wnt/PCP pathway [42, 46, 48–50].

It is unclear whether *Drosophila* DAAM participates in the establishment of PCP in the wing or eye, but it was demonstrated that DAAM regulates the actin cytoskeleton in the tracheal cuticle and the axonal growth cone [51, 52]. *Drosophila* DAAM interacts genetically with RhoA and Rac GTPases [51, 52], suggesting that Daam proteins have conserved functions as effectors of Rho GTPases. To determine whether Daam proteins function in the mammalian Wnt/PCP pathway, we generated loss-of-function (LOF) mutations in *Daam1* and *2*. We found that neither *Daam1* nor *Daam2* mutants display phenotypes characteristic of PCP mutants. However, genetic interactions between *Daam1* and *Wnt5a* or *Vangl2* are consistent with Daam1 having a role in the noncanonical Wnt pathway. Of note, the *Daam1* mutant had abnormal connections between maternal blood sinuses and fetal blood vessels in the placenta, and *Daam1/2* double mutants had a more severe phenotype. Our results demonstrate that Daam genes are essential genes with redundantly functions in placental development.

## Materials and methods

### Mice

*Daam1*^*Flox*^ *and Daam2*^*LacZ*^ mice were reported previously [53]. *Daam1* ^*FloxNeo/+*^heterozygotes were crossed with ACTB-flpe to remove the PGK-neo cassette. The resultant *Daam1*^*Flox/+*^ mice were crossed with ACTB-cre to generate the *Daam1* mutant allele (*Daam1*^*Δ/+*^). The *Daam1* mutant allele with PGK-neo cassette (*Daam1*^*ΔNeo/+*^) was generated by *Daam1*^*FloxNeo/+*^ heterozygotes crossed with ACTB-cre to remove the Exon6.

*Vangl2*^*Lp/+*^, ACT-flpe and Meox2-Cre mice were purchased from Jackson Labs. C57BL6 (Ly5.1 and Ly5.2) mice were provided by Charles River Laboratories. The *Wnt5a* knockout [54] and ACNB-cre [55] mice were obtained from the originating labs.

This study was carried out in compliance with the Guide for the Care and Use of Laboratory Animals of the National Institutes of Health. The protocol was approved by Frederick National Lab Animal Care and Use Committee (Proposal #17–408). Rodents were euthanized by CO_2_ inhalation in accordance with the most recent AVMA Guidelines on Euthanasia. For the harvesting of viable embryonic tissue, the embryos were harvested, placed in cold PBS, cooled down on ice and decapitated before harvesting of fetal livers.

### Antibodies and immunofluorescent reagents

The following antibodies and reagents were used: mouse anti-Daam1 C monoclonal antibodies were raised against mouse Daam1 peptide a.a.1057-1068 following standard procedures. Cloned lines were selected by ELISA and Western blotting and anti-Daam1 C monoclonal antibodies were purified from identified cell lines using a protein A column. Additional antibodies and reagents: mouse anti-human Daam1 N (a.a. 1–111) monoclonal antibody (M05) (Abnova), mouse anti-Actin monoclonal antibody (Chemicon), mouse anti-Myc (9E10) (Santa Cruz Biotechnology), rat anti-TER119-PE and rat anti-CD71-FITC (BD-Pharmingen), anti-PECAM-1 (MEC13.3)(BD-Pharmingen), goat anti-mouse Ig Horseradish Peroxidase-linked (GE healthcare), goat anti-rat Ig Horseradish Peroxidase-linked (GE healthcare), Rhodamine-phalloidin, Texas Red-X phalloidin, BODIPY488-phalloidin, and 4’,6-diamidini-2-phenylindole, dihydrochloride (DAPI) (BD Bioscience).

### Western blot

Whole embryonic day 10.5 (E10.5) embryos were dissected in cold PBS. Placentas were dissected and separated from the maternal portion. These samples were subsequently homogenized on ice in TNE (50 mM Tris-HCl (pH 7.5), 0.15 M NaCl, 1 mM EDTA, and 1% NP-40 with protease inhibitors Complete Mini (Roche)), centrifuged, and the supernatants were used. Protein concentrations were measured using the Bio-Rad Protein Assay (Bio-Rad) according to the manufacturer’s protocol. Proteins were separated using 4–15% Ready Gel (Bio-Rad), transferred to Immobilon transfer membrane (MILLIPORE), incubated with antibodies, and detected using the ECL Plus Western Blotting Detection System (GE Healthcare).

### Immunocytochemistry

Immunocytochemistry and stress fiber quantification were performed as previously described [46]. Briefly, the plasmids were transfected into NIH3T3 cells using Polyfect reagent (Qiagen). The cells were fixed, permeabilized, and stained with anti-Myc antibody and phalloidin. For quantification of effects on actin fibers, a baseline of ten fibers/cell was used. Cells containing greater or less than ten fibers were scored as a normal or depleted stress fiber, respectively.

### *Xenopus* embryo manipulation

Microinjection and manipulation of *Xenopus* embryos were performed as described previously [46]. Embryos with a curved and shortened axis were scored as mild defective embryos and embryos with an open neural tube and curved shortened axis were scored as severely defective embryos. The collective total number of injected embryos from all experiments is indicated below each bar.

### Flow cytometry, BFU-E colony formation assay and transplantation

Single-cell suspensions of fetal liver cells were collected from E12.5 embryos. For flow cytometry, cells were suspended in sort buffer (PBS (Gibco), penicillin/streptomycin (Gibco), and 0.2% de-ionized BSA (Sigma)), blocked with the non-specific FcR (CD16/32) with 2.4G2 (Purified anti-CD16/32: BD Phamingen), stained with antibodies, and analyzed with LSRI (Becton Dickinson).

For the BFU-E colony formation assay, 3 × 10^4^ cells were cultured for 10 days in IMDM (Gibco) with 1.1% Methylcellulose, 25% FCS, 30ng/ml IL-3, 100ng/ml SCF, 5 units/ml of Erythropoietin, 50 μM 2-Mercaptoethanol (SIGMA), and Penicillin/Streptomycin (Gibco). For transplantation, Ly5.2-positive C57BL6 mice were irradiated with 950rads, and 8× 10^5^ cells were injected intravenously. Transplanted mice were observed for two months, followed by harvesting of their thymus, spleen, and bone marrow for flow cytometry.

### Whole-mount immunohistochemistry

Embryos were fixed with 4%PFA/PBS and stained as previously described [56].

### Isolation of primary mouse embryonic fibroblasts (MEFs)

MEFs were prepared from E12.5 embryos by an established procedure [57]. Cell proliferation was measured using <4-passage cells in 96 well plates using the CellTiter96 Aqueous One Solution Cell proliferation Assay (MTS assay) (Promega) according to the manufacturer’s protocol.

### Reverse Transcription and quantitative PCR (qPCR)

Total RNA was purified using TRIzol (Invitrogen) according to the manufacturer’s recommendations. One microgram of RNA was treated with Dnase1 and first strand cDNA was synthesized using oligo dT and superscript III reverse transcriptase (Invitrogen). cDNA was quantitated by qPCR using the CFX96 real-Time PCR Detection System (Bio-Rad) and Fast Start Universal SYBR Green Master (Roche). The specificity of all primers was monitored by electrophoresis of the amplicons on agarose gels. The mean expression values obtained for each gene were normalized to GAPDH (ΔΔC (t) method) and to the expression of Daam1 in the lowest expressing sample. The following gene specific oligos were used.

Daam1-RT exon6 FW-CCAgAAgTATgCCAgCgAgAgAA

Daam1-RT exon7 RV-ACgCCCAgTgCTTTTATCCAAgT

GAPDH-RT.FW- AATgTgTCCgTCgTggATCTg;

GAPDH-RT.RV- CTgCTTCACCACCTTCTTgATgT.

### In situ hybridization

Section in situ hybridization was performed as previously described [47, 58]. Unless indicated otherwise, at least 4 mutant embryos were examined for each probe, and all yielded similar results.

### Histology

Embryos and placentas were dissected in ice-cold PBS and fixed with 4% PFA/PBS overnight at 4°C and transferred to 70% ethanol/saline for paraffin sections or 20% sucrose/PBS for frozen sections. Paraffin and frozen sections were cut at 8-μm and 10-μm thickness, respectively. Sections were stained with Hematoxylin and Eosin (H&E) following standard protocols.

### Plasmids

Daam1 vectors were constructed by standard restriction digestion or PCR amplification cloning techniques, and subcloned into the pCS2+MT vector. The plasmids designated as Daam1 FL, Daam1 N, and Daam1 Δ contain cDNA fragments that correspond to a.a.1-1077, 1–420, and 1–148 of the mouse Daam1 protein, respectively.

### GST pull-down assay

Myc-tagged proteins used in GST pull-down assays were generated by TNT Quick-Coupled Transcription/Translation systems (Promega). GST pull-down assays were performed as described previously [46].

### Imaging

Embryos and sections were photographed with a Leica MZFLIII stereoscope (Leica) or a Zeiss AxioPlan2, equipped with a Zeiss Axiocam HR digital camera, and Zeiss Axiovision version 4.5 imaging software (Zeiss).

### Statistical analysis

Student t-test and chi-square test were performed using excel software (Microsoft). Wilcoxon Rank-sum test was performed using R software (The R Foundation).

## Results

### Characterization of *Daam1* mutant mice

To address Daam1 function in vivo, we generated the *Daam1* null allele (*Daam1*^*Δ*^) from *Daam1* conditional knockout mice. Heterozygous *Daam1*^*Δ/+*^ mice are viable, fertile, and have no obvious phenotypes. Homozygous *Daam1*^*Δ/Δ*^ offspring arising from *Daam1*^*Δ/+*^ intercrosses were not found at birth (Table 1). Western blot analysis of whole embryo lysates using an antibody against the C-terminus of Daam1 confirmed that *Daam1*^*Δ/Δ*^ embryos did not express the full-length Daam1 protein, and that heterozygotes expressed roughly half the amount (S1A Fig). Further analysis of mutant embryos with an antibody that recognizes the N-terminus of Daam1 revealed a weakly expressed 16-kDa protein in *Daam1*^*Δ/Δ*^ lysates, which is the expected size when exon 6 of *Daam1* is removed from the locus (S1B Fig). Functional analysis of this 16-kDa Daam1Δ protein expressed in NIH3T3 and Xenopus embryos revealed it to be non-functional (S2 Fig). We therefore concluded that the *Daam1*^*Δ*^ allele is functionally null.

**Table 1:** Number of *Daam1*^*+/+*^, *Daam1*^*Δ/+*^, and *Daam1*^*Δ/Δ*^ for each developmental stage.

	*Daam1*^*+/+*^	*Daam1*^*Δ/+*^	*Daam1*^*Δ/Δ*^	Empty decidua
E10.5	9 (1)	29 (3)	18 (5)	4
E12.5	55 (1)	105 (2)	39 (10)	22
E13.5	25 (1)	60 (0)	14 (5)	17
E14.5	17 (1)	27 (2)	11 (6)	19
E16.5	15 (0)	22 (1)	2 (2)	1
Postnatal	29	89	0	

Numbers of dead embryos are indicated in parentheses.

**Fig 1:**
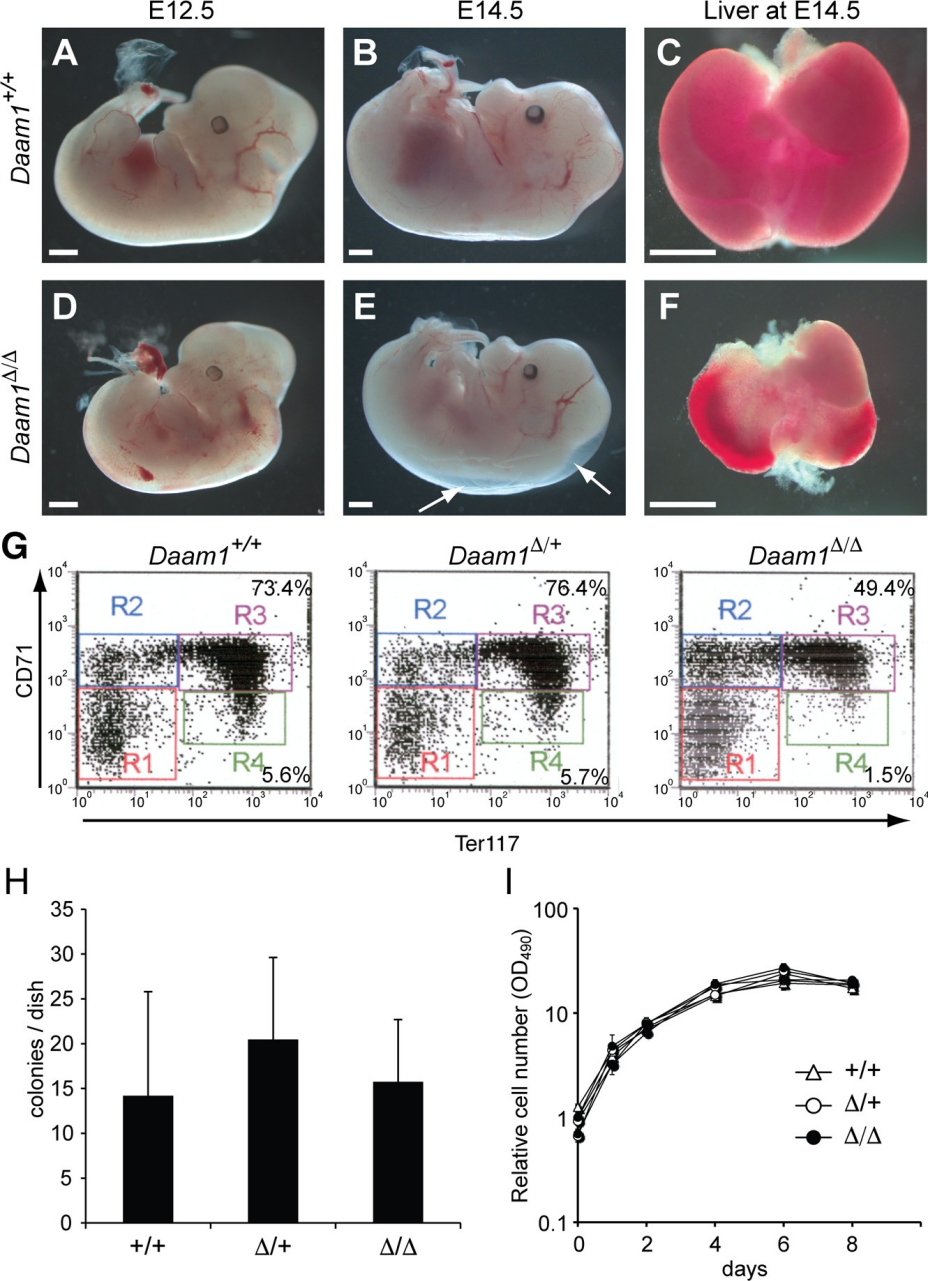
Observation of *Daam1*-deficient embryos. (A-E) *Daam1*^+/+^ (A-C) and *Daam1*^*Δ /Δ*^ (D-F) embryos at E12.5 (A, D), E14.5 (B, E) and livers at E14.5 (C, F). Edema found in *Daam1*^*Δ /Δ*^ embryo is indicated by arrows (E). (G) Representative results of fetal liver-derived erythrocyte differentiation analyzed by flow cytometry. Percentages of the cells gated in R3 and R4 were as follows: *Daam1*^+/+^: R3 (CD71^hi^;Ter119^hi^) = 76.5 ± 2.4%, R4 (CD71^lo^;Ter119^hi^) = 6.4 ± 0.8%; n = 5, *Daam1*^*Δ/+*^ R3 = 73.8 ± 6.8%, R4 = 6.3 ± 1.7%; n = 4, and *Daam*^*Δ /Δ*^:R3 = 64.2 ± 9.0%, R4 = 3.3 ± 1.6%; n = 5. *Daam1*^*Δ /Δ*^ erythrocytes were significantly reduced compared with *Daam1*^+/+^ and *Daam1*^*Δ/+*^: t-test R3 p<0.01, R4 p<0.002. (H) BFU-E colony formation assay using fetal liver cells. *Daam1*^+/+^: n = 5, *Daam1*^*Δ/+*^: n = 5, and *Daam1*^*Δ/Δ*^: n = 4. Standard deviations are shown as error bars. No significant difference was observed. (I) MEFs proliferation assay. Relative cell numbers were measured by MTS assay. Results were normalized by day 0 as 1. Results represent analyses of 5 or more embryos of each genotype. No significant difference was observed.

Analysis of *Daam1*^*Δ/Δ*^ embryos demonstrated that embryo viability declined after E12.5, and live embryos were no longer observed by E16.5 (Table 1). E12.5–14.5 *Daam1*^*Δ/Δ*^ embryos were small and pale compared with wild-type and *Daam1*^*Δ/+*^ littermates (Fig 1A, 1B, 1D and 1E). Reduced liver mass was apparent in whole *Daam1*^*Δ/Δ*^ embryos by E14.5 (Fig 1C and 1F), but reduced numbers of total fetal liver cells were quantified as early as E12.5 (2.2-fold reduction) and E13.5 (4-fold reduction) compared with wild-type and *Daam1*^*Δ/+*^ littermates. The differentiation status of hematopoietic cells of the mutant fetal liver was assessed by flow cytometry. *Daam1*^*Δ/Δ*^ embryos exhibited normal differentiation of the T cell, B cell, and myeloid cell lineages, however erythrocyte differentiation was perturbed. Using CD71 and Ter119 expression as markers of erythrocyte differentiation [59], we found that *Daam1*^*Δ/Δ*^ embryos had fewer differentiated, mature erythrocyte cells, than wild-type and *Daam1*^*Δ/+*^ embryos at E12.5 (Fig 1G). The reduction of mature erythrocytes may have been caused by fewer erythrocyte progenitors or an inability to differentiate. To examine these possibilities, we performed an erythroid burst-forming units (BFU-E) colony formation assay. *Daam1*^*Δ/Δ*^ and control embryos had similar numbers of erythrocyte progenitors (Fig 1H). To examine their differentiation capacity *in vivo*, fetal liver cells from wild-type and mutant embryos were transplanted into lethally irradiated wild-type hosts. Engraftment of *Daam1*^*Δ/Δ*^ fetal liver cells leading to the viability of recipient hosts was observed (S1 Table), demonstrating that *Daam1*^*Δ/Δ*^ hematopoietic progenitor cells retain the ability to differentiate into functional erythrocytes. These results suggest that the reduced viability of *Daam1*^*Δ/Δ*^ embryos was not due to intrinsic defects in erythropoiesis or fetal liver function.

### Delayed development of *Daam1*^*Δ/Δ*^ mutants due to placental defects

As *Daam1*^*Δ/Δ*^ embryos were smaller than their littermates, we considered the possibility that Daam1 might regulates cell proliferation. MEFs were established from E12.5 embryos, and cell proliferation was analyzed by MTS assay. No difference was observed in the number of *Daam1*^*Δ/Δ*^ MEFs compared with wild-type and *Daam1*^*Δ/+*^ MEFs (Fig 1I), indicating that Daam1 does not play a major role in cell proliferation.

**Fig 2:**
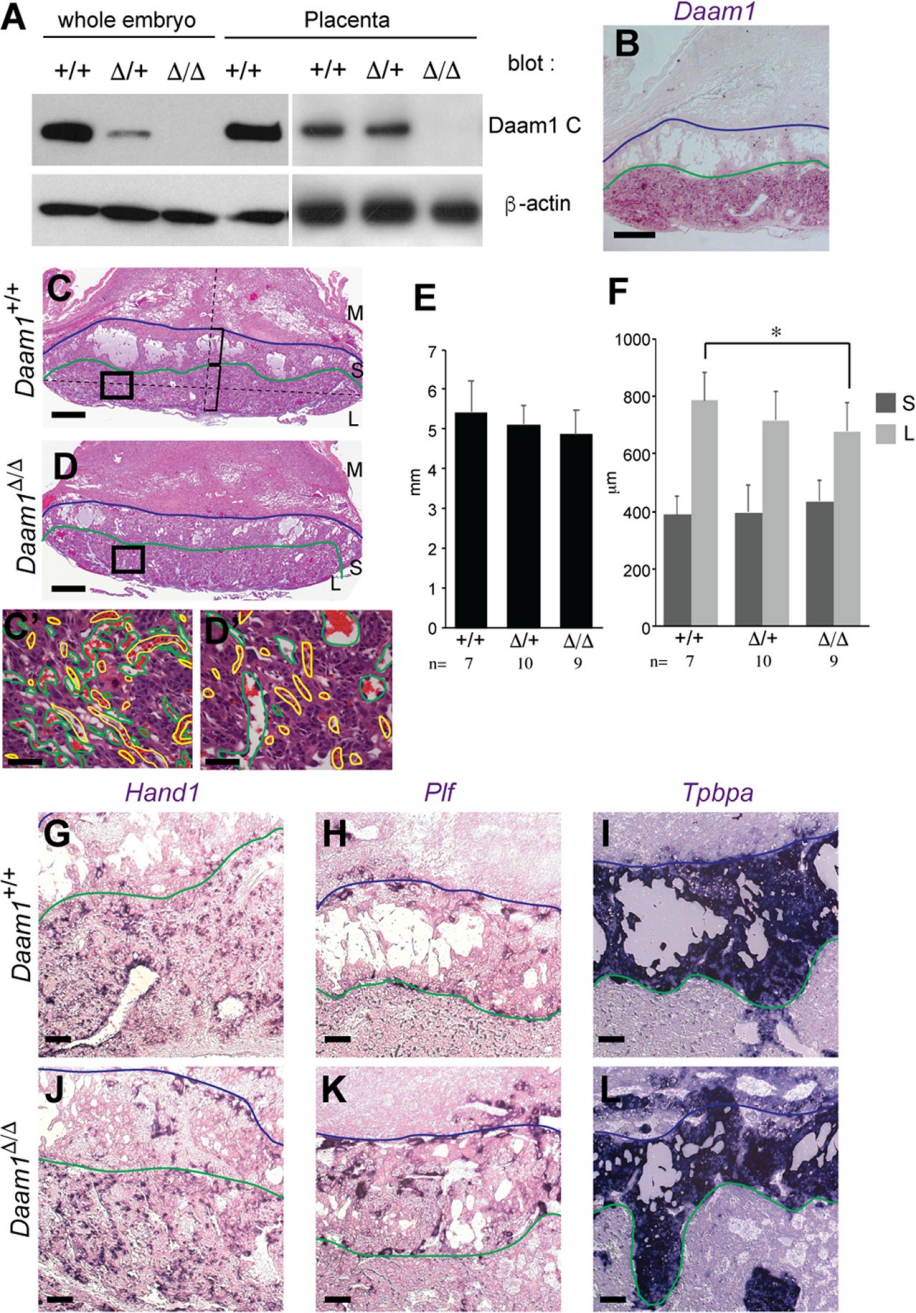
Placental developmental defects in *Daam1*-deficient mice. (A) Western blot analysis for Daam1 (top) and β-actin (bottom). Whole embryo lysates and the embryonic part of the placenta from indicated genotypes at E10.5 were used.(B) In situ hybridization of wild-type placenta sections using the *Daam1* probe. (C) *Daam1*^+/+^ and (D) *Daam1*^*Δ/Δ*^ placentas at E12.5. High magnification images’ (C’, D’) positions are indicated as boxes on C and D. Maternal blood sinuses (green) and fetal blood vessels (yellow) are labeled with lines on C’ and D’. (E) Size of the placenta of each genotype which was measured as the distance between edges of the boundary between the spongiotrophoblast and labyrinthine layers. (F) Thickness of the spongiotrophoblast layer (S) and labyrinthine layer (L) in each genotype. The thickness of each layers was measured on the line that connected the center of the boundary between spongiotrophoblast and labyrinthine layers and the thickest part of placenta as shown in C. t-test *: p<0.05. (G-L) In situ hybridization of *Daam1*^+/+^ (G-I) and *Daam1*^*Δ/Δ*^ (J-L) placenta sections using *Hand1* (G, J), *Plf* (H, K) and *Tpbpa* (I, L) probes. Blue lines depict the boundary between the maternal decidua (M) and spongiotrophoblast layer (S), and green lines depict the boundary between spongiotrophoblast and labyrinthine layers (L). Scale bar = 200 μm in B, and G-L. 500 μm in C and D. 50 μm in C’ and D’.

In addition to the reduced size of *Daam1*^*Δ/Δ*^ embryos, we also observed edema, which is indicative of a circulatory defect (Fig 1E). Circulatory defects can arise from defects in the vasculature, the heart, or the placenta. As the vasculature had developed normally in *Daam1*^*ΔNeo/ΔNeo*^ embryos (S3 Fig), we considered a role of Daam1 in heart and placental function. As reported in a previous paper [53], myocardial-specific *Daam1* conditional KO exhibits myocardial maturation defects, but does not cause embryonic lethality. Analysis of Daam1 protein expression in the placenta by Western blotting confirmed that Daam1 was indeed expressed in the placenta, at a similar level to that observed in embryos, and was undetectable in the *Daam1*^*Δ/Δ*^ placenta (Fig 2A). In situ hybridization of sectioned placenta revealed that *Daam1* mRNA was highly expressed in the placenta, although restricted to the labyrinthine layer (Fig 2B). The labyrinthine layer plays an important role in placental function, because it is where the direct exchange of nutrition and gases between the maternal and fetal blood supplies occurs [60]. Defects in the function of the labyrinthine layer lead to placental insufficiency, which may account for the fetal growth retardation and death. Histological analyses revealed that the spongiotrophoblast layer in *Daam1*^*Δ/Δ*^ placentas were of a similar size and thickness as controls, but the labyrinthine layer in *Daam1*^*Δ/Δ*^ placentas was slightly thinner than in wildtype. (Fig 2C–2F).

Marker analyses using the trophoblast giant cell marker *Hand1*, the endothelial trophoblast marker *Plf*, and the spongiotrophoblast marker *Tpbpa*, revealed no differences in their expression between control and *Daam1*^*Δ/Δ*^ placentas (Fig 2G–2L) suggesting that cell fates were unchanged in *Daam1*^*Δ/Δ*^ placentas. A detailed analysis of the labyrinthine layers revealed thin and refined maternal blood sinuses adjacent to fetal blood vessels in control placentas, however, maternal blood sinuses were enlarged and not entwined with fetal blood vessels in the *Daam1*^*Δ/Δ*^ labyrinthine layer (Fig 2C’ and 2D’). These results suggest that nutritional deficiency caused by reduced placental function was responsible for the delayed development of Daam1 mutants.

To assess whether *Daam1* has an intrinsic function in the embryo, we used *Meox2*-cre to inactivate *Daam1* specifically in the epiblast, thereby enabling wild-type function in the placenta. As *Daam1* and *Meox2* are located on the same chromosome, mutant alleles (*Daam1*^*Flox/Δ*^; *Meox2-cre*) on different sister chromosomes were rarely inherited together (Table 2). Notably, *Daam1*^*Flox/Δ*^; *Meox2-cre* mice were viable for >1 year and lacked overt phenotypes (Table 2). These results are consistent with the absence of intrinsic function by Daam1 during embryonic development, however, the mosaic expression of *Meox2-cre* in the epiblast [61] remains a caveat. Indeed, *Daam1* RNA and protein remain detectable in varying amounts in adult organs, depending on the tissue (ex. 1.3–29.6% of *Daam1* RNA level observed in control tissues (S4A and S4B Fig)). Nevertheless, these results together suggest that the embryo lethality of *Daam1*^*Δ/Δ*^ mutants is due to the requirement of Daam1 in the placenta.

**Table 2:** Number of embryos or mice from crosses between *Daam1*^*flox/flox*^ and *Daam1*^*Δ/+*^*; Meox2-cre* mice.

	*Daam1*^*Flox/+*^	*Daam1*^*Flox/+*^*; Meox2-cre*	*Daam1*^*Flox/Δ*^	*Daam1*^*Flox/Δ*^*; Meox2-cre*
E16.5	4	18	12	3
Postnatal	3	36	40	3

### Daam1 and Daam2 are functionally redundant in the placenta

Despite the broad pattern of embryonic expression [47], removal of Daam1 only resulted in placental defects. To address the possibility that Daam2 functionally compensates for the loss of Daam1 in the embryo, we used *Daam2*^*LacZ*^ mice. Examination of β-galactosidase activity in *Daam2* heterozygous (*Daam2*^*LacZ/*+^) embryos revealed a pattern similar to that of endogenous *Daam2* mRNA expression (S5A Fig, and [47]). *Daam2*^*LacZ/LacZ*^mice were also viable and fertile and had no obvious phenotype.

**Fig 3:**
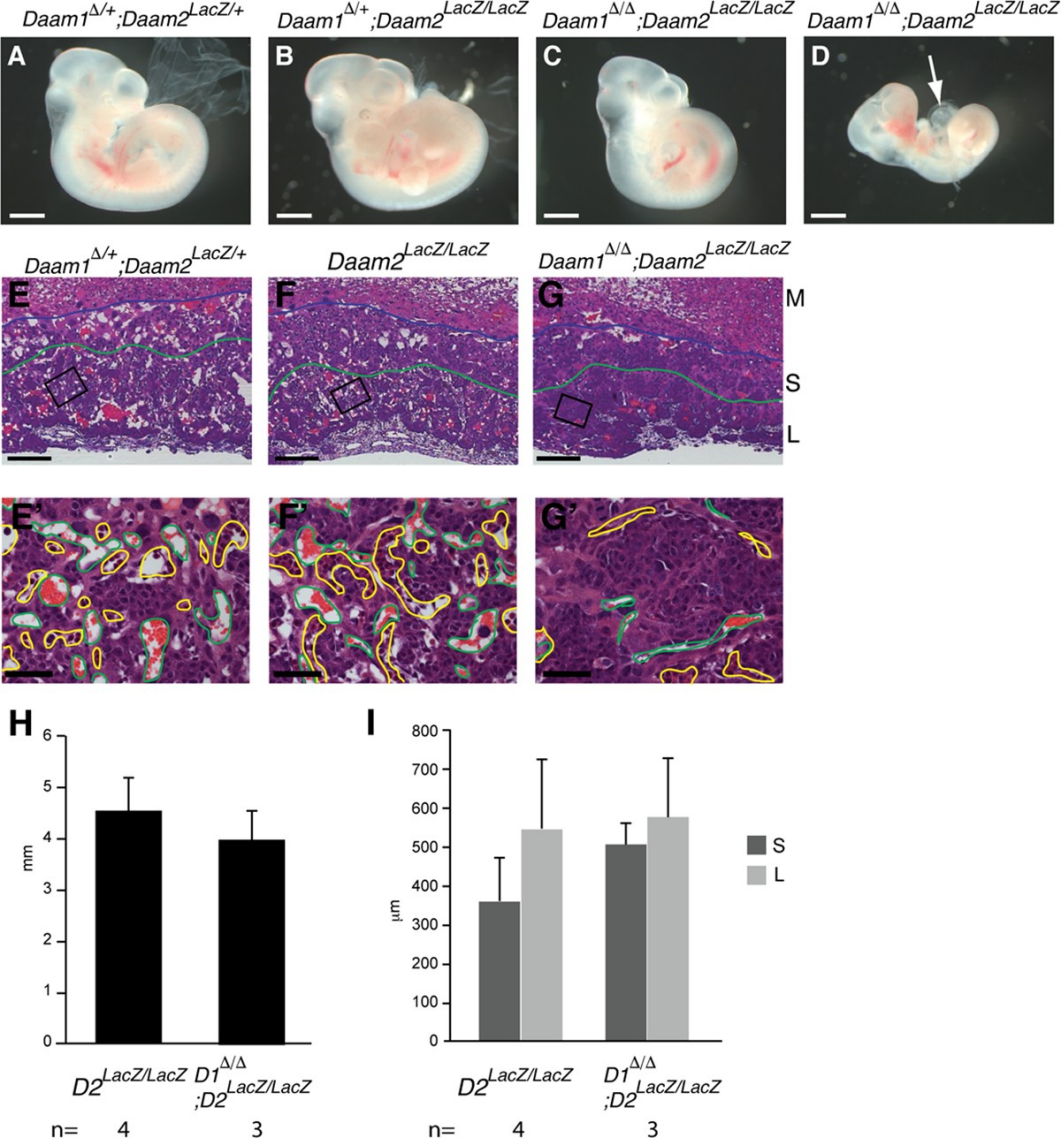
Embryonic developmental delay and placental developmental defects in *Daam1/2*-deficient mice. (A-C) *Daam1*^*Δ/+*^, *Daam2*^*LacZ/+*^ (A), *Daam1*^*Δ/+*^, *Daam2* ^*LacZ/LacZ*^ (B), and *Daam1*^*Δ/Δ*^, *Daam2*^*LacZ/LacZ*^ (C, D), embryos at E10.5. Some of *Daam1*^*Δ/Δ*^, *Daam2* ^*LacZ/LacZ*^ embryos were found dead with an enlarged pericardial cavity (arrow, D). (E-G, E’-G’) H&E staining of E10.5 placenta sections of *Daam1*^*Δ/+*^, *Daam2* ^*LacZ/+*^ (E), *Daam2* ^*LacZ/LacZ*^ (F), and *Daam1*^*Δ/Δ*^, *Daam2* ^*LacZ/LacZ*^ (G). High magnification images’ (E’-G’) positions are indicated as boxes on E-G. (H) The size of the placenta of *Daam2* ^*LacZ/LacZ*^ and *Daam1*^*Δ/Δ*^, *Daam2* ^*LacZ/LacZ*^ was measured as described for Fig 2E. (I) The thickness of the spongiotrophoblast layer (S) and labyrinthine layer (L) in *Daam2* ^*LacZ/LacZ*^ and *Daam1*^*Δ/Δ*^, *Daam2* ^*LacZ/LacZ*^, measured as described in Fig 2F. Blue lines depict the boundary between the maternal decidua (M) and spongiotrophoblast layer (S), and green lines depict the boundary between the spongiotrophoblast and labyrinthine layers (L). Maternal blood sinuses and fetal blood vessels are outlined in green and yellow, respectively (E’-G’). Scale bars = 1 mm in A-D, 200 μm in E-G, 50 μm in E’- G’.

On examination of *Daam1*^*Δ/Δ*^*;Daam2*^*LacZ/LacZ*^ embryos at E10.5, double mutants were observed to be smaller than their litter mates (n = 8/16) (Fig 3A–3D) and had enlarged pericardial cavities (n = 3/16) (Fig 3D). All the *Daam1*^*Δ/Δ*^*;Daam2* ^*LacZ/LacZ*^ embryos dissected at E11.5 (n = 4) and E12.5 (n = 2), and the *Daam1*^*Δ/Δ*^*;Daam2* ^*LacZ/+*^ embryos dissected at E12.5 (n = 5) were dead and had started to be resorbed (Table 3). Daam2 expression in placenta was examined by β-gal staining. Daam2 was highly expressed in embryonic mesoderm-derived tissue, and expressed at low levels in the labyrinthine layer, but not in the spongiotrophoblast (S5B and S5B’ Fig). Examination of *Daam1*^*Δ/Δ*^*;Daam2* ^*LacZ/LacZ*^ placentas at E10.5, revealed normal fusion of the chorioallantoic mesoderm in controls and *Daam1*^*Δ/Δ*^*;Daam2* ^*LacZ/LacZ*^ placentas (Fig 3E–3G and 3E’–3G’), but vascularization of the labyrinthine layer was markedly impaired in *Daam1*^*Δ/Δ*^*;Daam2* ^*LacZ/LacZ*^ placentas (Fig 3G and 3G’). As these placental defects were more severe and occured earlier (E10.5) in the double mutants than in the single *Daam1*^*Δ/Δ*^ placentas (E12.5) (Figs 2C’ and 2D’ and 3E‘–3G’), we conclude that Daam1 and Daam2 have redundant functions in placental development.

**Table 3:** Number of *Daam1* and *Daam2* mutants for each developmental stage.

	*Daam1*^*+/+*^*;Daam2*^*+/+*^	*Daam1*^*+/+*^*; Daam2*^*LacZ/+*^	*Daam1*^*+/+*^*; Daam2*^*LacZ/LacZ*^	*Daam1*^*Δ/+*^*; Daam2*^*+/+*^	*Daam1*^*Δ/+*^*; Daam2*^*LacZ/+*^	*Daam1*^*Δ/+*^*; Daam2*^*LacZ/LacZ*^	*Daam1*^*Δ/Δ*^*; Daam2*^*+/+*^	*Daam1*^*Δ/Δ*^*; Daam2*^*LacZ/+*^	*Daam1*^*Δ/Δ*^*; Daam2*^*LacZ/LacZ*^
E10.5	1	8	20	9	18	33 (1)	1	12 (2)	16 (2)
E11.5			4			11			4 (4)
E12.5	2	2	12	2	10	9	0	5 (5)	2 (2)

E10.5 and E12.5 embryos were obtained from *inter se* crosses of *Daam1*^*Δ/+*^, *Daam2* ^*LacZ/+*^ or *Daam1*^*Δ/+*^, *Daam2* ^*LacZ/LacZ*^ mice.

E11.5 embryos were obtained from *inter se* crosses of *Daam1*^*Δ/+*^, *Daam2* ^*LacZ/LacZ*^ mice.

Numbers of dead embryos are indicated in parentheses.

### Genetic interaction between Daam1 and PCP pathway components

**Fig 4:**
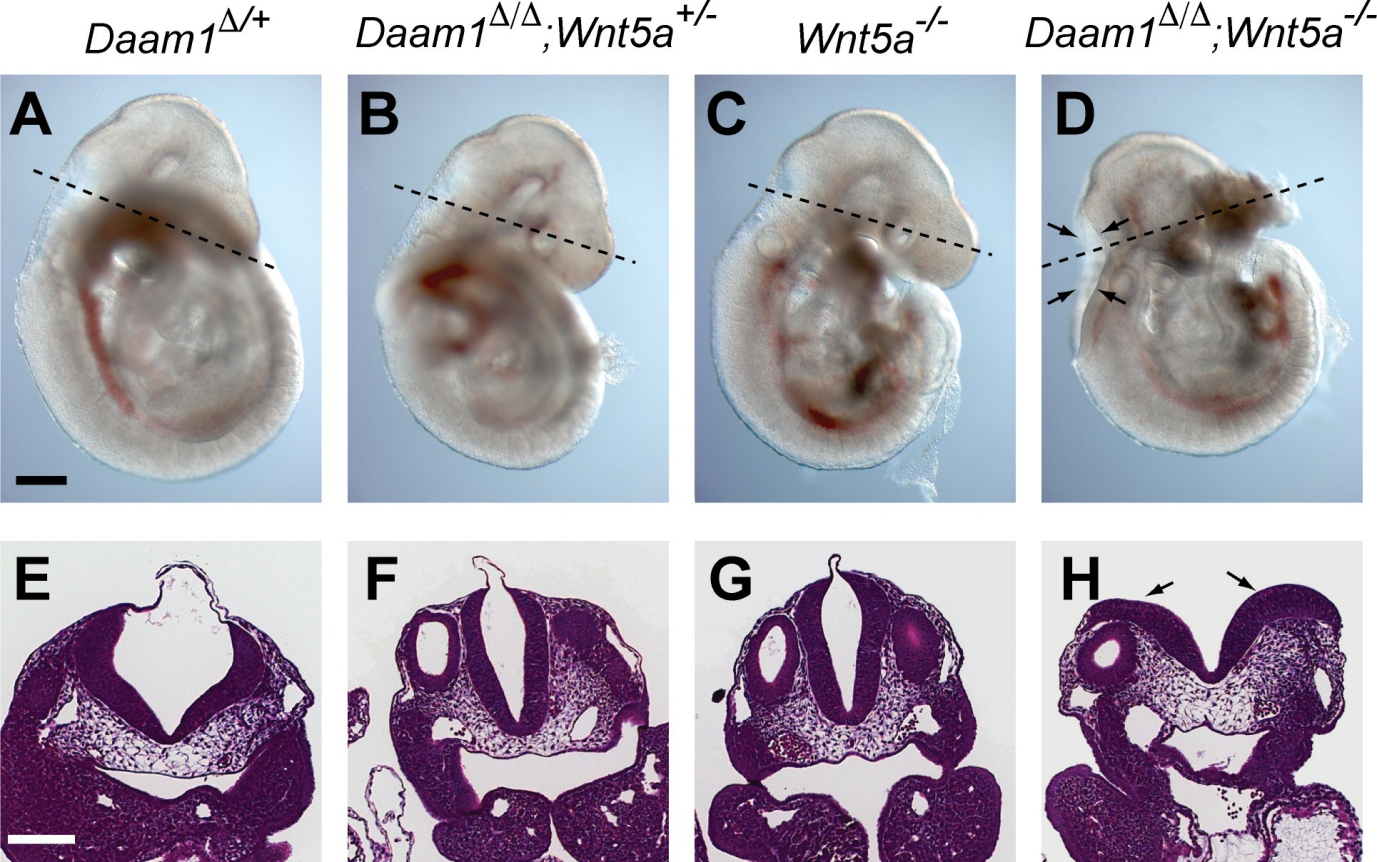
Neural tube closure defects in *Daam1*;*Wnt5a* double mutants. (A-D) Lateral view of (A) *Daam1*^*Δ/+*^, (B) *Daam1*^*Δ/Δ*^, *Wnt5a*^*+/-*^, (C) *Wnt5a*^*-/-*^ and (D) *Daam1*^*Δ/Δ*^, *Wnt5a*^*-/-*^ embryos at E9.5. H&E staining of cross-sections of the embryos shown in A-D through the fourth ventricle (E-H) Section positions are indicated by dotted lines in A-D. Arrows indicate the open neural tube. Scale bars = 200 μm.

Although Daam1 was originally identified as a regulator of Wnt/PCP signaling [42], phenotypes characteristic of mammalian PCP mutants such as *Vangl2*^*Lpt*^ such as neural tube closure defects and convergent-extension defects, were not observed in Daam mutants. To investigate whether Daam1 has a role in the mammalian PCP pathway, we looked for genetic interactions between *Daam1* and *Vangl2* or *Wnt5a* mutants, both of which are components of the noncanonical PCP pathway [24, 54, 62, 63]. Vaginal atresia or imperforate vagina is a little-discussed but frequently observed phenotype of *Vangl2*^*Lpt*^ mice [63], including our colony of *Vangl2*^*Lpt/+*^ females (7 of 15 mice). This frequency increased when crossed with the *Daam1*^*Δ/+*^ background (13 of 15 *Daam1*^*Δ/+*^;*Vangl2*^*Lpt/+*^ females). However, vaginal atresia in *Daam1*^*Δ/+*^;*Vangl2*^*Lpt/+*^ female mice precluded the generation of *Daam1*^*Δ/Δ*^;*Vangl2*^*Lpt/Lpt*^ embryos. Examination of *Daam1* and *Wnt5a* interactions at E9.5 (Table 4) revealed that *Daam1*^*Δ/ Δ*^*;Wnt5a*^*+/-*^ embryos were indistinguishable from wild-type, *Daam1*^*Δ/+*^, or D*aam1*^*Δ/ Δ*^ embryos (Fig 4A, 4B, 4E and 4F). Although *Daam1*^*Δ/ Δ*^*;Wnt5a*^*-/-*^ embryos exhibited a short AP axis and small somite phenotype similar to *Wnt5a*^*-/-*^ embryos (Fig 4C and 4D) [54], double mutants frequently exhibited an open neural tube (5 of 8 *Daam1*^*Δ/ Δ*^*;Wnt5a*^*-/-*^embryos) at the position of the fourth ventricle (Fig 4D and 4H). This phenotype was never observed in *Daam1*^*Δ/ Δ*^ or *Wnt5a*^*-/-*^ single mutants.

**Table 4:** Number of *Daam1* and *Wnt5a* mutants at E9.5 stage.

*Daam1*^*+/+*^*; Wnt5a*^*+/+*^	*Daam1*^*+/+*^*; Wnt5a*^*+/-*^	*Daam1*^*+/+*^*; Wnt5a*^*-/-*^	*Daam1*^*Δ/+*^*; Wnt5a*^*+/+*^	*Daam1*^*Δ/+*^*; Daam2*^*LacZ/+*^	*Daam1*^*Δ/+*^*; Daam2*^*LacZ/LacZ*^	*Daam1*^*Δ/Δ*^*; Wnt5a*^*+/+*^	*Daam1*^*Δ/Δ*^*; Wnt5a*^*+/-*^	*Daam1*^*Δ/Δ*^*; Wnt5a*^*-/-*^
13	27	10	22	46	11	7	11	8

These embryos were obtained from 15 litters of *inter se* crosses of *Daam1*^*Δ/+*^, *Wnt5a*^*+/-*^, 1 litter of *Daam1*^*Δ/+*^, *Wnt5a*^*+/-*^ male crossed with *Wnt5a*^*+/-*^ female, and 4 litter of *Daam1*^*Δ/+*^, *Wnt5a*^*+/-*^ male crossed with *Daam1*^*Δ/+*^ females.

## Discussion

We investigated the role of Daam1 in mammalian development and discovered that Daam1 plays an essential role in murine placentation. The placenta functions as a fetomaternal organ during gestation, enabling nutrient uptake, waste elimination, and gas exchange between the mother and developing conceptus. Most rodents and primates (including humans) have hemochorial or labyrinthine placentas, in which maternal blood directly contacts the fetal chorionic trophoblast. The close apposition of the vascularized uterus and trophoblast in the labyrinth constitutes the major site of maternal and fetal exchange. We demonstrated that *Daam1* is expressed in the trophoblast cells of the labyrinthine layer, but not in the embryonic mesoderm-derived vascular cells, and that *Daam1*^*Δ/Δ*^ placentas undergo normal trophoblast cell differentiation but exhibit structural defects in the labyrinthine layer. As gas and nutrient exchange relies upon diffusion across the placental membrane (reviewed in [64, 65]), we suggest that the increased separation between maternal blood sinuses and fetal blood vessels observed in the *Daam1*^*Δ/Δ*^ labyrinthine layer (Fig 2D’) is sufficient to impair the efficiency of the maternal/fetal exchange, and is therefore likely to result in fetal death. The observation that the embryo-specific deletion of *Daam1* did not impair fetal development is consistent with a requisite role for Daam1 in placental trophoblast cells. How does Daam1 control the maternal blood sinus and fetal blood vessel connection? One possible explanation is that Daam1 controls trophoblast branching morphogenesis in the labyrinthine layer which is essential for the vascularization of the labyrinth, as the Wnt/PCP genes have been reported to be involved in branching morphogenesis of other epithelial tissues [66–69]. Another possibility is that Daam1 controls the maternal blood sinus and fetal blood vessel connection by stabilizing cell-cell junctions. Daam1 interacts with the E-cadherin–β-catenin–α-catenin complex at the lateral membrane contact sites to stabilize the interactions between epithelial cells [70]. Similar molecular mechanisms might operate at the maternal blood sinus and fetal blood vessel connection. Indeed, the absence of E-cadherin or β-catenin in the placenta also causes vascularization defects in the labyrinthine layers [71, 72].

Embryos lacking both *Daam1* and *Daam2* died earlier than single *Daam1* mutants, but they did not have additional embryonic phenotypes. *Daam1*^*Δ/Δ*^, *Daam2* ^*LacZ/LacZ*^ placentas had more marked vascularization defects than *Daam1*^*Δ/Δ*^ placentas suggesting that the earlier embryo lethality arises from the more severe placental defects. mDia1, a paralog of Daam1/2, was reported to interact with RhoA, B, and/or C to control actin assembly, similar to Daam1 [73, 74]: reviewed in [45, 75]. *mDia1* deficient mice also had no developmental defects [76], raising the possibility that these family members have redundant functions. Future studies are needed to address whether mDia1, Daam1 and Daam2 compensate for one another.

Our data demonstrating that the reduced *Daam1* gene dosage worsened the vaginal atresia phenotype in *Vangl2*^*Lpt/+*^ mice and caused NTDs in *Wnt5a*^*-/-*^ embryos. Most of the NTDs reported in the PCP core factors mutant mice were craniorachischisis, complete open neural tube [20, 21, 28, 29, 34–36, 62], however the NTD observed in *Daam1*^*Δ/ Δ*^*;Wnt5a*^*-/-*^ embryos was opened only at the hindbrain. Similar partial NTDs were observed compound heterozygous of PCP core factor mutants [35] and Dvl2 single null mice [21] with low penetrance. It is likely that the partial NTDs are mild phenotype of NTDs caused by the PCP pathway aberration. The NTD observed in *Daam1*^*Δ/ Δ*^*;Wnt5a*^*-/-*^ embryos suggested that Daam1 functioning, at least in part, in the Wnt/PCP pathway. This is supported by a report demonstrating a key role for Daam1 in the Wnt/PCP pathway during chick neural tube closure [77]. However, neither *Daam1*^*Δ/Δ*^ nor *Daam1*^*Δ/Δ*^, *Daam2* ^*LacZ/LacZ*^ mouse embryos exhibited neural tube closure defects and the early embryonic lethality observed in *Daam1*^*Δ/Δ*^, *Daam2* ^*LacZ/LacZ*^ embryos precludes our ability to fully evaluate the role of the *Daam1* gene in the PCP pathway. A report of hypomorphic *Daam1* mutants, which survive to later developmental stages, described cardiac outflow tract septation defects that are commonly found in PCP mutants [78]. As severe circulation defects from placental abnormality in *Daam1*^*Δ/Δ*^ and *Daam1*^*Δ/Δ*^, *Daam2* ^*LacZ/LacZ*^ mouse embryos caused midgestational lethality, we were unable to assess heart developmental defects in Daam1 and Daam2 null mutants. Our previous study of cardiomyocyte-specific conditional *Daam1* knockouts revealed indispensable roles of Daam1 in cardiac development [53]. We did not observe heart defects in *Daam1*^*Flox/Δ*^; *Meox2-cre* mice presumably due to leaky expression of Daam1 caused by the mosaic expression of Meox2-cre. The low frequency of surviving *Daam1*^*Flox/Δ*^; *Meox2-cre* adult mice (3 out of 83 mice) compared with the frequency of *Daam1*^*Flox/Δ*^; *Meox2-cre* embryos (3 out of 37 embryos) suggest that some mutants did not survive to adulthood.

In addition to the neural tube and cardiac outflow tract, the Wnt/PCP pathway was reported to regulate tube formation in the kidney, gut, and female reproductive tract [25, 79–84]. Tissue-specific inactivation of *Daam1* and *Daam2* in these organs will provide an opportunity to analyze the role of Daam proteins in these PCP-dependent tissues at later developmental stages.

A role for the PCP pathway in placental development has not been previously described. However, mice deficient in *Wnt2* or the Wnt receptor *Fz5* were found to have vascularization defects in the labyrinthine layer of the placenta similar to the *Daam1/2* mutant phenotype [85, 86]. *Wnt2* is expressed in embryo-derived allantoic mesoderm cells, whereas *Fz5*, like *Daam1*, is expressed in the extraembryonic labyrinth layer. During vascularization, interactions between trophoblast and embryonic mesoderm cells are important for forming capillary structures. We speculate that Wnt ligands provided by the allantois stimulate trophoblast cells in a paracrine manner to regulate capillary formation. Of note, Wnt2 is known to regulate the Wnt/β-catenin pathway in the developing lung [87], and Daam2 participates in the dorsal patterning of the spinal cord via the Wnt/β-catenin pathway [88]. Together, these results suggest that Daam1 and Daam2 could function in different Wnt pathways in a tissue-dependent manner. Another possibility is that Daam1 and Daam2 function in different signaling pathways to control placental development. Studies on Daam1 and Daam2 functions in cardiomyocytes and endothelial cells [53, 89] suggested that Daam1 has roles in the activation of the Src-Akt-Gsk3β pathway. Indeed, mice deficient in *Akt1* also exhibit vascularization defects in the labyrinthine layer of the placenta that are similar to the *Daam1/2* mutant phenotype [90].

We demonstrated that Daam1 and Daam2 play essential roles in placental vascularization and the establishment of the maternal/fetal blood supply. Feto-placental problems are proposed to be one of the major causes of pre-eclampsia, intrauterine growth restriction and miscarriage [91]. Further analysis of the mechanisms through which Daam functions in the placenta may improve our understanding of placental pathologies.

## Supporting information

S1 Fig Daam1 protein expression in *Daam1* knock out mice. Western blot analysis to confirm Daam1 expression. (A) The top panel shows the results using an antibody against the Daam1 C-terminus antigen. (B) Top panels show the results using an antibody against the Daam1 N-terminus antigen. The right panel is longer exposure of the left panel. The arrow and arrowhead indicate full length and truncated Daam1 protein, respectively. The asterisk indicates non-specific bands. The bands labeled by double asterisks are likely degraded or splicing variants of Daam1 protein. Bottom panels show actin as loading controls.
(TIF)

S2 Fig Daam1Δ allele product interacts with RhoA but does not have dominant-negative activity. (A) GST-pulldown assay of Daam1 truncated proteins with GST-RhoA. Top panels showed mouse Daam1 Δ, Daam1 N (dominant-negative form), and Daam1 FL (full length) proteins from left to right, detected by anti-Myc antibody. Bottom panels show GST or GST-RhoA proteins detected by Coomassie staining. Daam1 Δ transfected cells (B-D) and Daam1 N transfected cells (E-G) are shown. (B, E) Daam1 truncated proteins were detected by anti-Myc antibody. (C, F) Phalloidin stained cells. (C’, F’) High-magnification image of the inset is shown on the side. (D. G) Merged images of B, C and E, F are shown. (H) Quantification of effects by overexpression of Daam1 deletion proteins on stress fibers. Examined cell numbers are indicated below the graph. Chi-square test *: p<0.001 (I) Xenopus embryos were injected with mRNA transcribed from indicated plasmids, and were scored at stage 35. Scoring was performed following previously described criteria [46] Examined embryo numbers are indicated below the graph. Wilcoxon Rank-sum test *: p<0.001 (J) Representative embryos injected with each mRNA are shown.
(TIF)

S3 Fig Analysis of vasculature development in *Daam1* knock out mice. PECAM-1 staining of Daam1^+/+^(A), Daam1^ΔNeo/+^(B), and Daam1^ΔNeo/ΔNeo^ embryos at E10.5 stage are shown. No gross abnormalities in vasculature development were observed in these embryos nor in the *Daam1*^*Δ/Δ*^embryos.
(TIF)

S4 Fig Expression of *Daam1* in *Daam1*^*Flox/Δ*^; *Meox2-cre* mice. (A) Expression of *Daam1* was examined by qPCR. Relative *Daam1* expression in each tissue is shown. Mut-4(*Daam1*^*F/Δ*^*;Meox2-cre*), and cont-1 (*Daam1*^*F/Δ*^), mut-5 (*Daam1*^*F/Δ*^*;Meox2-cre*), and cont-2 (*Daam1*^*F/+*^), mut-6 (*Daam1*^*F/Δ*^*;Meox2-cre*), and cont-3 (*Daam1*^*F/+*^*;Meox2-cre*) are litter mates, respectively. SEM is shown as error bar. Western blot analysis for Daam1 protein (top) and β-actin (bottom). Pedigree number and tissues are shown above and below the panels, respectively.
(TIF)

S5 Fig Analysis of β-galactosidase reporter expression in *Daam2*^*LacZ*^ embryos. (A) X-gal staining of E10.5 *Daam2*^*LacZ/+*^ embryo. (B) X-gal staining of E10.5 placental section. High magnification image (B’) positions are indicated as boxes on E. Arrow indicates embryo-derived mesodermal tissue. Blue and green lines depict the boundary between the maternal decidua (M) and spongiotrophoblast layer (S), and the spongiotrophoblast and labyrinthine layers (L), respectively. Scale bars = 500 μm in D, 200 μm in E, and 50 μm in E.
(TIF)

S6 Fig Raw images of Fig 2A and S4 Fig. (TIF)

S7 Fig Raw images of Fig 2A. (TIF)

S8 Fig Raw images of S1 Fig. (TIF)

S9 Fig Raw images of S2A Fig. (TIFF)

S1 Table Transplantation of fetal liver cells into lethally irradiated recipient mice. (DOCX)
