# Supplementary figures and images for: Placental defects lead to embryonic lethality in mice lacking the Formin and PCP proteins Daam1 and Daam2
Source: PLoS One. 2020 Apr 30;15(4):e0232025. doi: 10.1371/journal.pone.0232025 (PMC7192421; doi:10.1371/journal.pone.0232025)

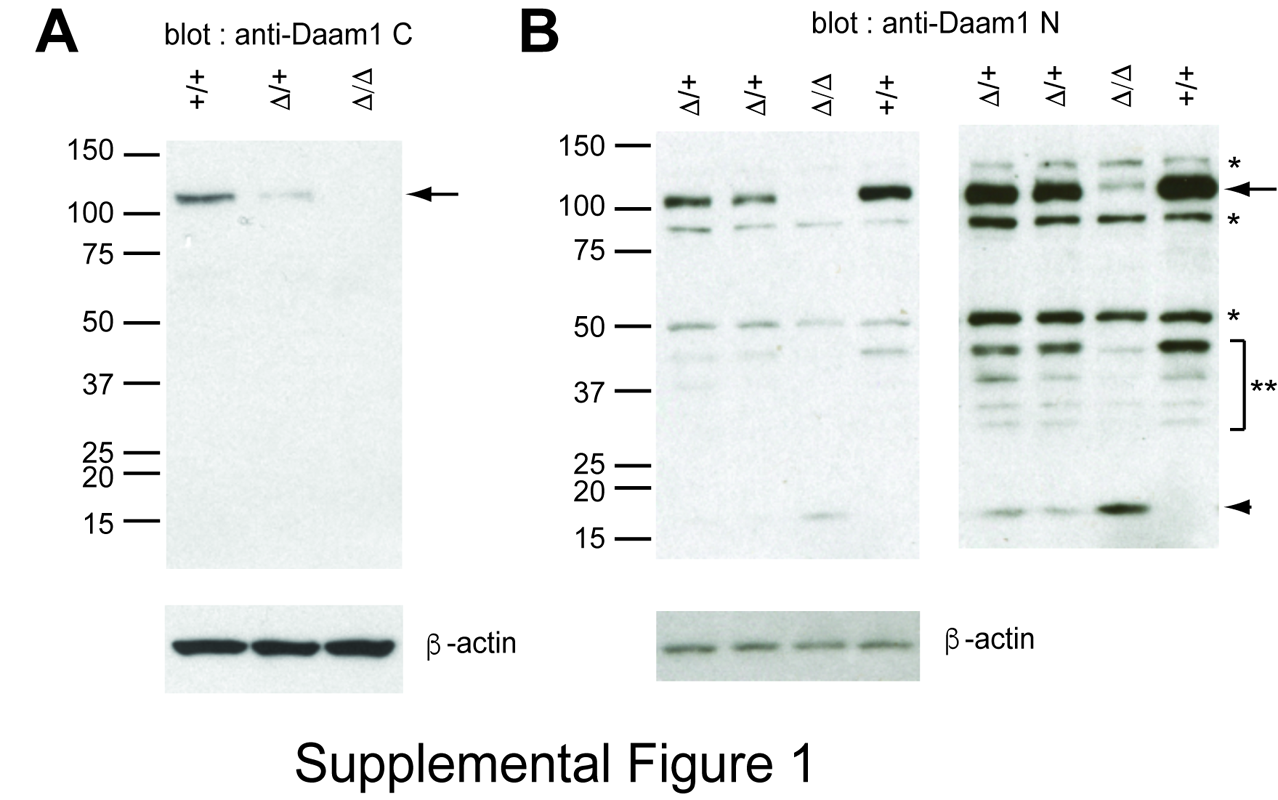

Supplement: S1 Fig — Western blot analysis to confirm Daam1 expression. (A) The top panel shows the results using an antibody against the Daam1 C-terminus antigen. (B) Top panels show the results using an antibody against the Daam1 N-terminus antigen. The right panel is longer exposure of the left panel. The arrow and arrowhead indicate full length and truncated Daam1 protein, respectively. The asterisk indicates non-specific bands. The bands labeled by double asterisks are likely degraded or splicing variants of Daam1 protein. Bottom panels show actin as loading controls. (TIF) [file pone.0232025.s001.tif]

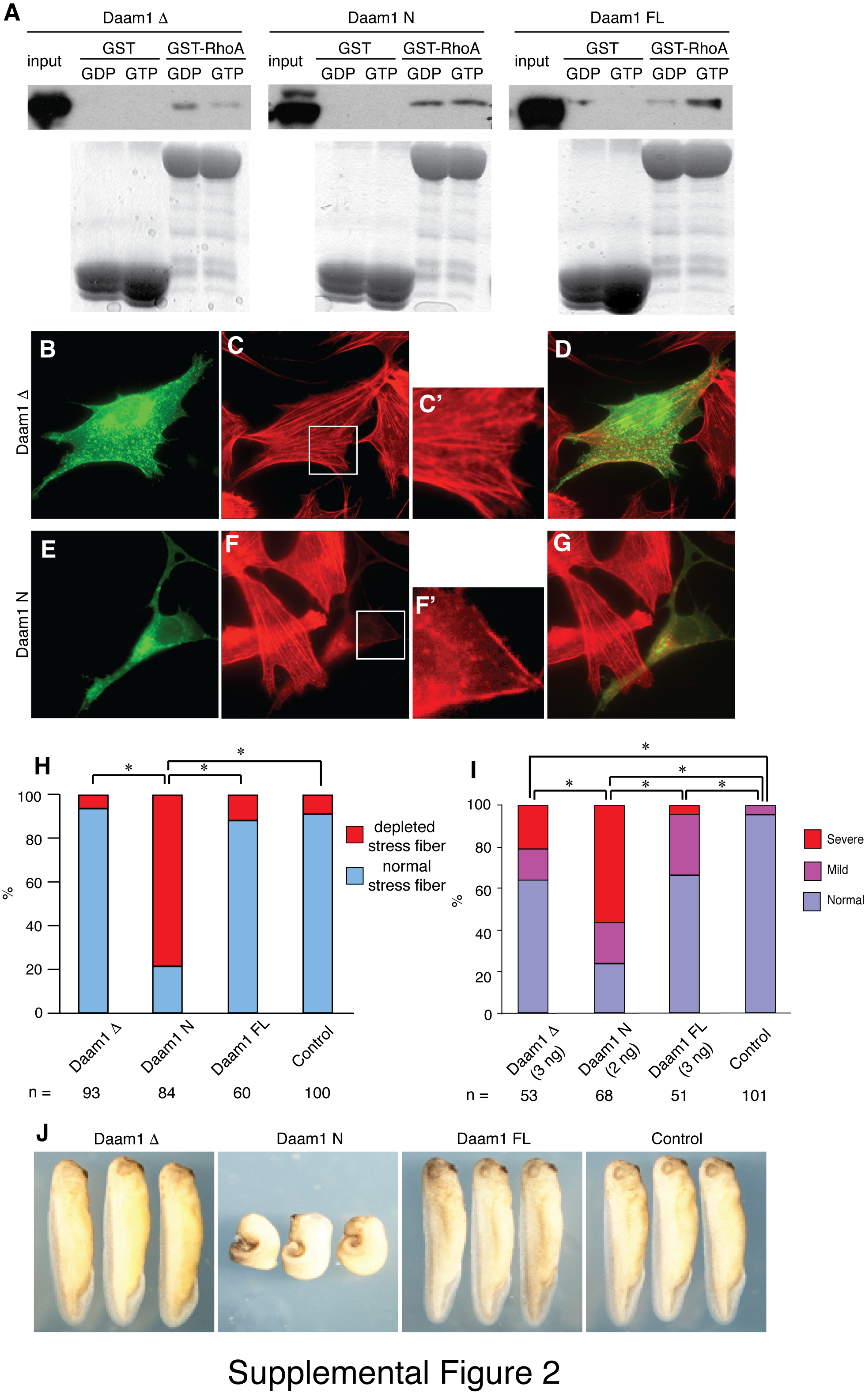

Supplement: S2 Fig — (A) GST-pulldown assay of Daam1 truncated proteins with GST-RhoA. Top panels showed mouse Daam1 Δ, Daam1 N (dominant-negative form), and Daam1 FL (full length) proteins from left to right, detected by anti-Myc antibody. Bottom panels show GST or GST-RhoA proteins detected by Coomassie staining. Daam1 Δ transfected cells (B-D) and Daam1 N transfected cells (E-G) are shown. (B, E) Daam1 truncated proteins were detected by anti-Myc antibody. (C, F) Phalloidin stained cells. (C’, F’) High-magnification image of the inset is shown on the side. (D. G) Merged images of B, C and E, F are shown. (H) Quantification of effects by overexpression of Daam1 deletion proteins on stress fibers. Examined cell numbers are indicated below the graph. Chi-square test *: p<0.001 (I) Xenopus embryos were injected with mRNA transcribed from indicated plasmids, and were scored at stage 35. Scoring was performed following previously described criteria [46] Examined embryo numbers are indicated below the graph. Wilcoxon Rank-sum test *: p<0.001 (J) Representative embryos injected with each mRNA are shown. (TIF) [file pone.0232025.s002.tif]

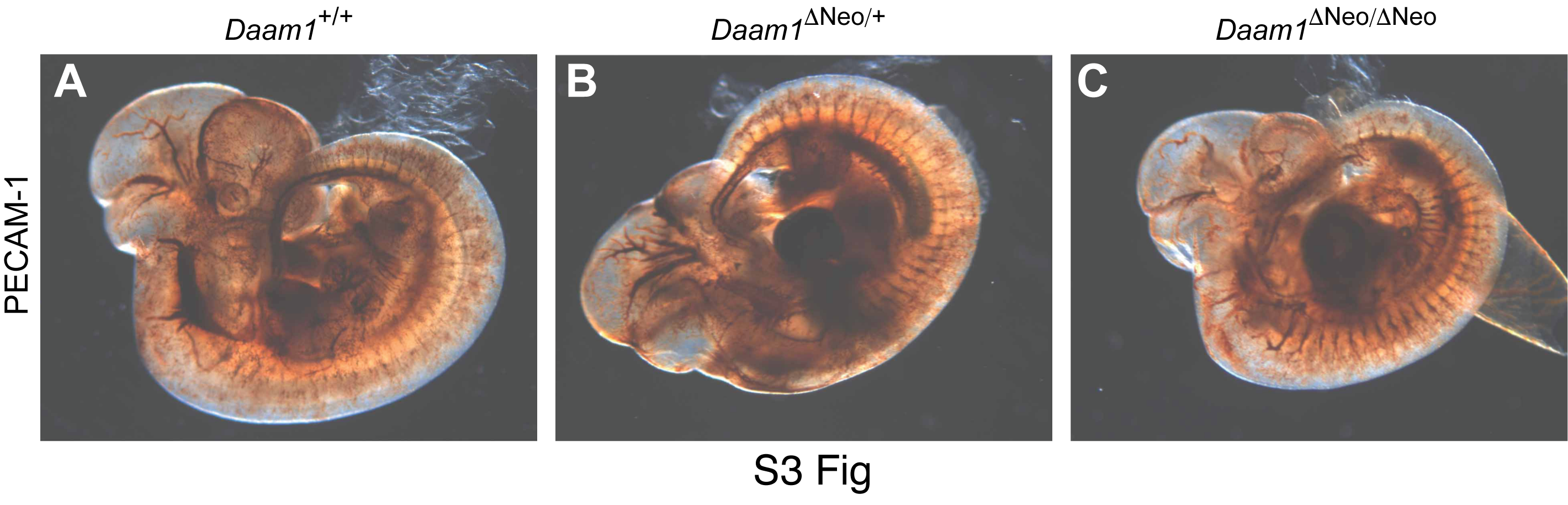

Supplement: S3 Fig — PECAM-1 staining of Daam1+/+(A), Daam1ΔNeo/+(B), and Daam1ΔNeo/ΔNeo embryos at E10.5 stage are shown. No gross abnormalities in vasculature development were observed in these embryos nor in the Daam1Δ/Δembryos. (TIF) [file pone.0232025.s003.tif]

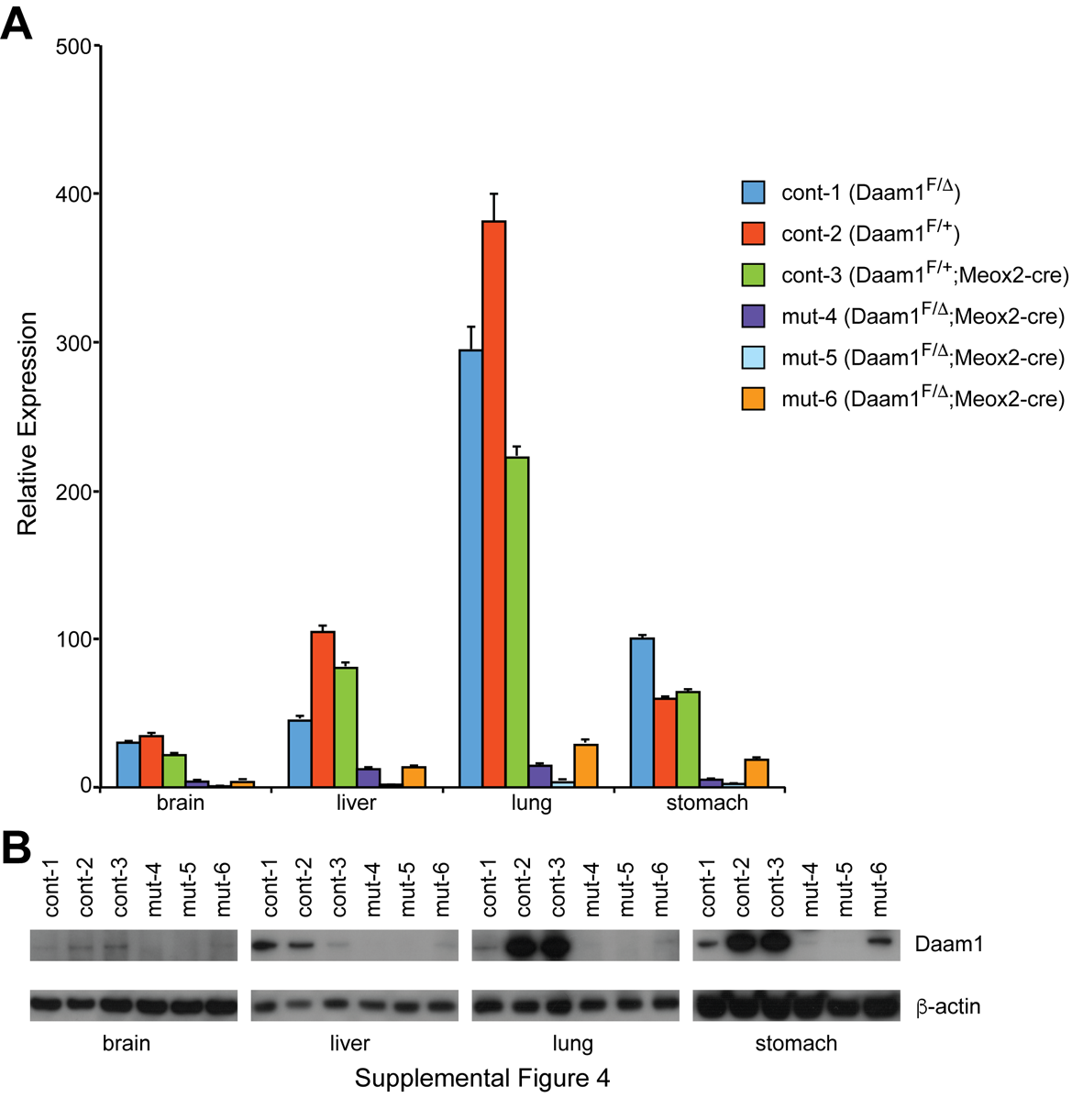

Supplement: S4 Fig — (A) Expression of Daam1 was examined by qPCR. Relative Daam1 expression in each tissue is shown. Mut-4(Daam1F/Δ;Meox2-cre), and cont-1 (Daam1F/Δ), mut-5 (Daam1F/Δ;Meox2-cre), and cont-2 (Daam1F/+), mut-6 (Daam1F/Δ;Meox2-cre), and cont-3 (Daam1F/+;Meox2-cre) are litter mates, respectively. SEM is shown as error bar. Western blot analysis for Daam1 protein (top) and β-actin (bottom). Pedigree number and tissues are shown above and below the panels, respectively. (TIF) [file pone.0232025.s004.tif]

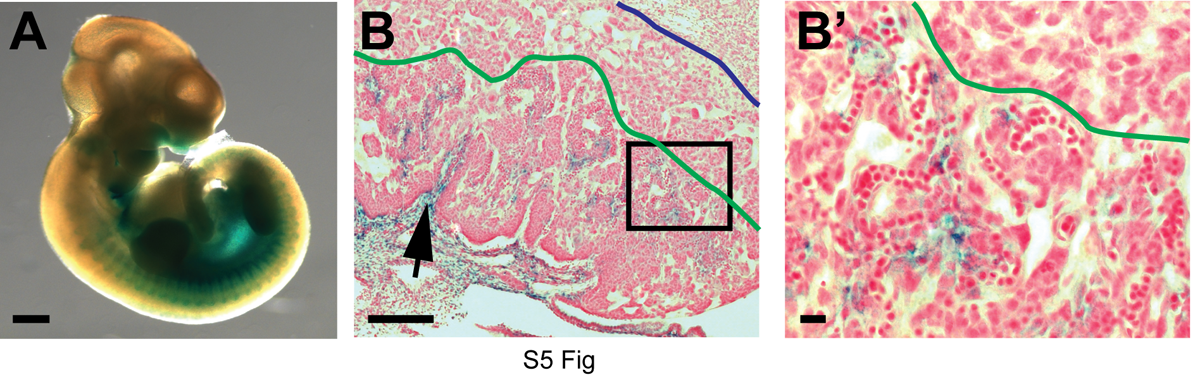

Supplement: S5 Fig — (A) X-gal staining of E10.5 Daam2LacZ/+ embryo. (B) X-gal staining of E10.5 placental section. High magnification image (B’) positions are indicated as boxes on E. Arrow indicates embryo-derived mesodermal tissue. Blue and green lines depict the boundary between the maternal decidua (M) and spongiotrophoblast layer (S), and the spongiotrophoblast and labyrinthine layers (L), respectively. Scale bars = 500 μm in D, 200 μm in E, and 50 μm in E. (TIF) [file pone.0232025.s005.tif]

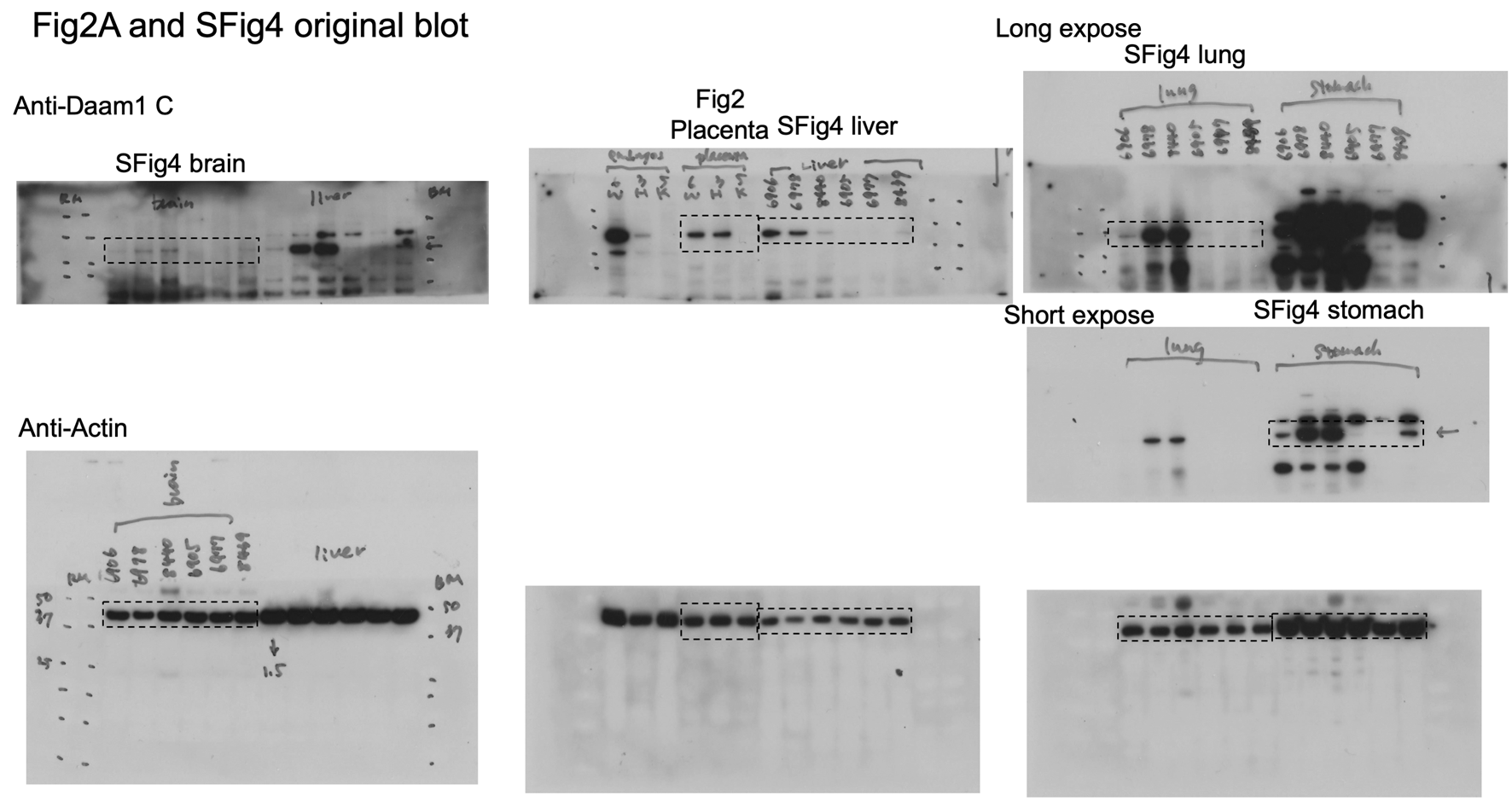

Supplement: S6 Fig — (TIF) [file pone.0232025.s006.tif]

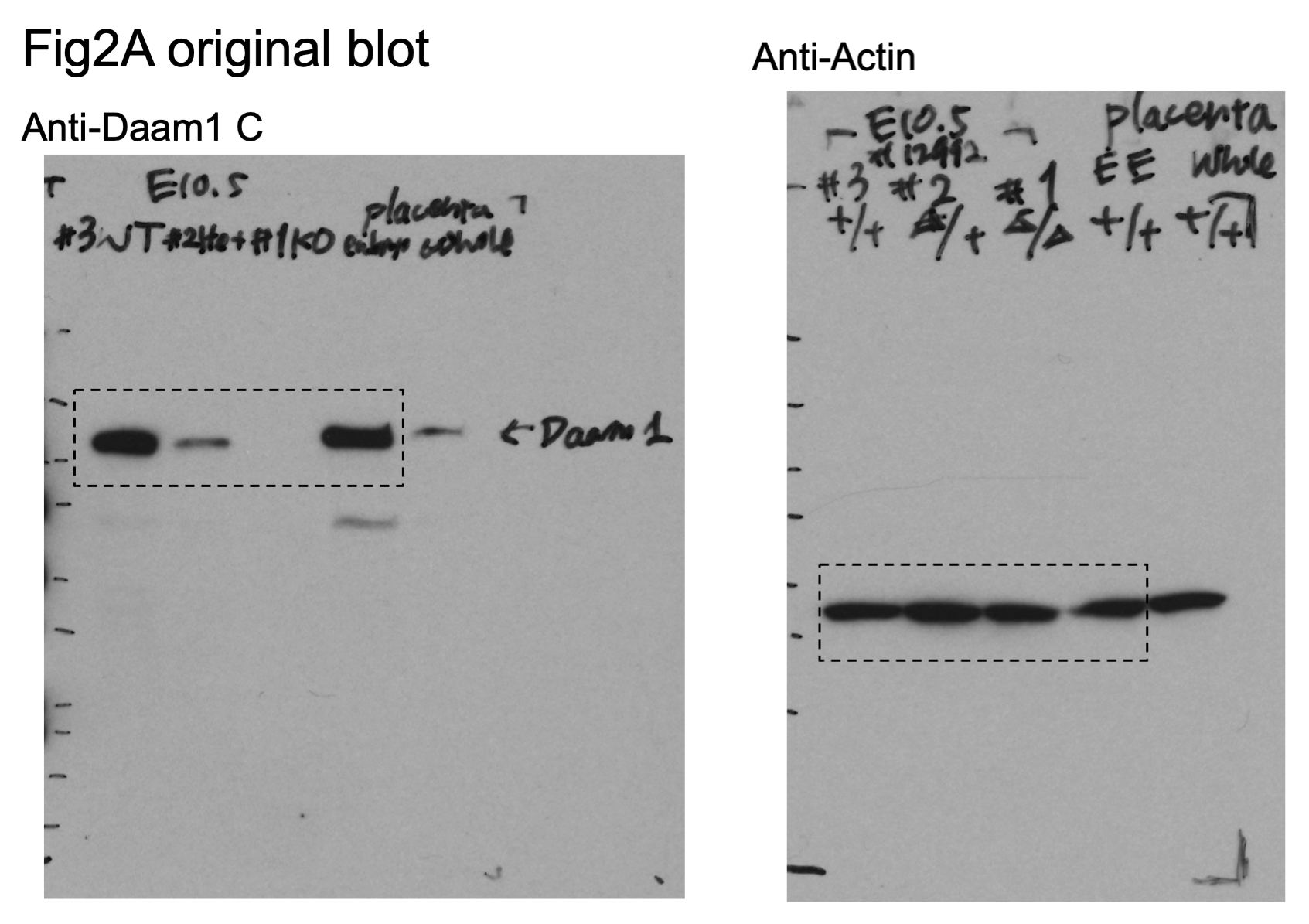

Supplement: S7 Fig — (TIF) [file pone.0232025.s007.tif]

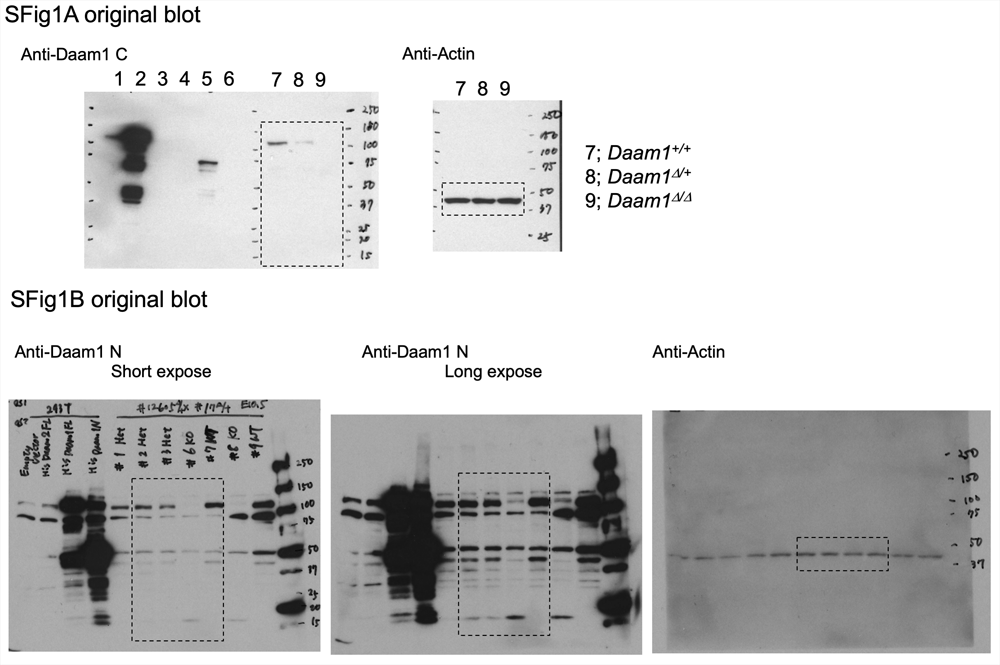

Supplement: S8 Fig — (TIF) [file pone.0232025.s008.tif]

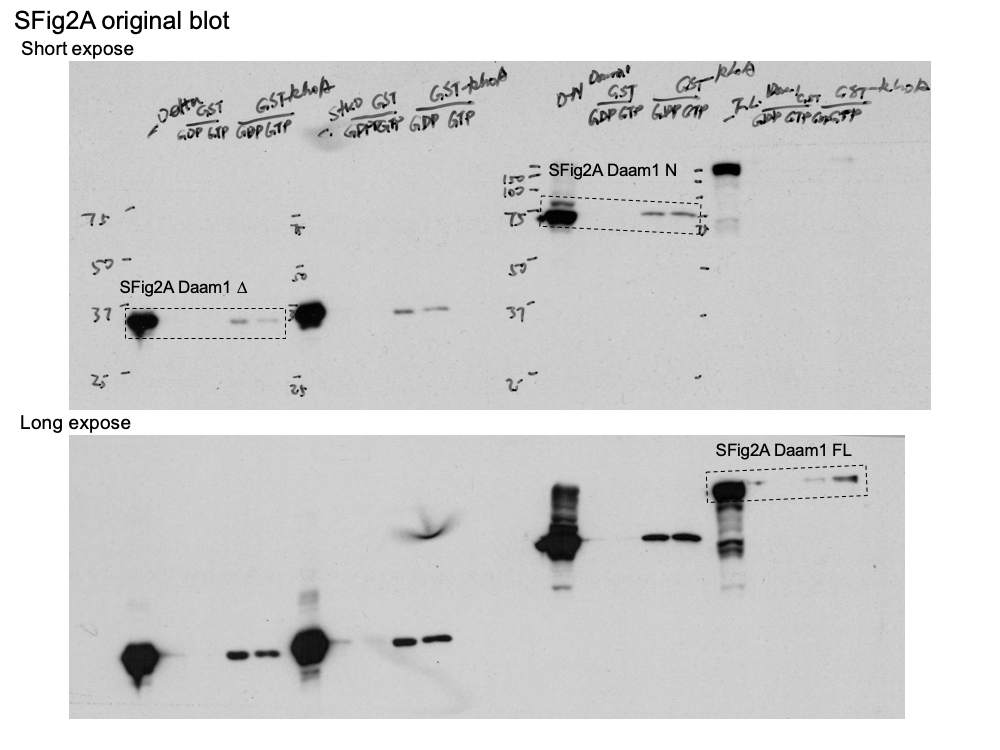

Supplement: S9 Fig — (TIFF) [file pone.0232025.s009.tiff]
