# Supplementary material for: Placental defects lead to embryonic lethality in mice lacking the Formin and PCP proteins Daam1 and Daam2
Source: PLoS One. 2020 Apr 30;15(4):e0232025. doi: 10.1371/journal.pone.0232025 (PMC7192421; doi:10.1371/journal.pone.0232025)
Supplement: S1 Table — (DOCX) [file pone.0232025.s010.docx]

**S1 Table. Transplantation of fetal liver cells into lethally irradiated recipient mice**

| Exp. | Donor | Recipient | No. mice | Engraftment |
| --- | --- | --- | --- | --- |
| 1 | control (Saline) | WT | 2 | 0/2 |
|  | +/+ | WT | 3 | 3/3 |
|  | Δ/+ | WT | 3 | 2/3 |
|  | Δ/Δ | WT | 3 | 3/3 |
| 2 | control (Saline) | WT | 2 | 0/2 |
|  | Δ/+ | WT | 5 | 5/5 |
|  | Δ/Δ | WT | 4 | 4/4 |
| 3 | control (Saline) | WT | 2 | 0/2 |
|  | +/+ | WT | 3 | 3/3 |
|  | Δ/+ | WT | 3 | 3/3 |
|  | Δ/Δ | WT | 3 | 3/3 |
